# Biclustering Methods: Biological Relevance and Application in Gene Expression Analysis

**DOI:** 10.1371/journal.pone.0090801

**Published:** 2014-03-20

**Authors:** Ali Oghabian, Sami Kilpinen, Sampsa Hautaniemi, Elena Czeizler

**Affiliations:** 1 Institute of Biotechnology, University of Helsinki, Helsinki, Finland; 2 Institute of Molecular Medicine for Finland (FIMM), University of Helsinki, Helsinki, Finland; 3 Institute of Biomedicine and Genome-Scale Biology Research Program, University of Helsinki, Helsinki, Finland; 4 Department of Computer Science and Engineering, Aalto University, Espoo, Finland; University of North Carolina at Charlotte, United States of America

## Abstract

DNA microarray technologies are used extensively to profile the expression levels of thousands of genes under various conditions, yielding extremely large data-matrices. Thus, analyzing this information and extracting biologically relevant knowledge becomes a considerable challenge. A classical approach for tackling this challenge is to use clustering (also known as one-way clustering) methods where genes (or respectively samples) are grouped together based on the similarity of their expression profiles across the set of all samples (or respectively genes). An alternative approach is to develop biclustering methods to identify local patterns in the data. These methods extract subgroups of genes that are co-expressed across only a subset of samples and may feature important biological or medical implications. In this study we evaluate 13 biclustering and 2 clustering (*k*-means and hierarchical) methods. We use several approaches to compare their performance on two real gene expression data sets. For this purpose we apply four evaluation measures in our analysis: (1) we examine how well the considered (bi)clustering methods differentiate various sample types; (2) we evaluate how well the groups of genes discovered by the (bi)clustering methods are annotated with similar Gene Ontology categories; (3) we evaluate the capability of the methods to differentiate genes that are known to be specific to the particular sample types we study and (4) we compare the running time of the algorithms. In the end, we conclude that as long as the samples are well defined and annotated, the contamination of the samples is limited, and the samples are well replicated, biclustering methods such as Plaid and SAMBA are useful for discovering relevant subsets of genes and samples.

## Introduction

Modern high-throughput measurement technologies, such as microarrays, are able to quantify expression levels for tens of thousands of genes in various organisms. One of the approaches for analysis and interpretation of large quantities of high-throughput data is clustering (also known as one-way clustering), where genes, samples, or both, are grouped together based on their gene expression profiles [1], [2]. For instance, Sørlie *et al.* analyzed gene expression data for 85 breast cancer samples with hierarchical clustering to suggest five subclasses for breast cancer [3].

Hierarchical clustering with heatmap visualization [4], *k*-means clustering and self-organizing maps [5], [6] have been successful in finding biologically important groups of genes or samples. These methods, however, do not take full advantage of the data as clustering is done first for genes and then for samples (or *vice versa*). Thus, groups of genes that are co-expressed only in a subset of samples may be left undetected. A promising solution to identify subgroups of genes and samples is the so called biclustering approach [7]. An important distinction between biclustering methods and one-way clustering methods, such as hierarchical clustering or *k*-means, is that the clustering is done simultaneously for genes and samples. Wang et al. [8] used a biclustering algorithm (CMonkey [9]) to group breast tumors from 437 individuals based on the expression profiles of specific genes. They reported that it is possible to identify co-expressed gene-sets in the subgroups of breast tumor samples using biclustering methods.

Given that the concept behind the biclustering approach is appealing in biosciences, a number of biclustering methods have been developed [10]–[13]. Here, we used two gene expression data to compare the performance of 13 biclustering and two clustering (*k*-means and hierarchical) methods. The first data comprises five different types of tissues consisting of expression data with heterogeneous samples that resides bicluster structures with small overlaps on their genes and samples. For the second data set we chose two clinically well-defined subgroups of breast tumor (ER+/PR+/HER2+ and ER−/PR−/HER2−) and reference breast samples. Due to the homogeneity of the samples and the common active biological pathways in different tumor subtypes, the breast cancer data is expected to reside bicluster structures with overlapping genes and samples. For our comparison analysis, we applied four benchmarks: Sample differentiation, Gene Ontology-based significance, Tissue specificity of the genes, and Running time.

## Materials and Methods

Biclustering methods can be categorized based on the type of the searched biclusters as well as the mathematical formulation used to discover them. Using these two criteria, we have categorized biclustering techniques into four classes: Correlation maximization biclustering methods, Variance minimization biclustering methods, Two-way clustering methods, and Probabilistic and generative methods.

**Figure 1:**
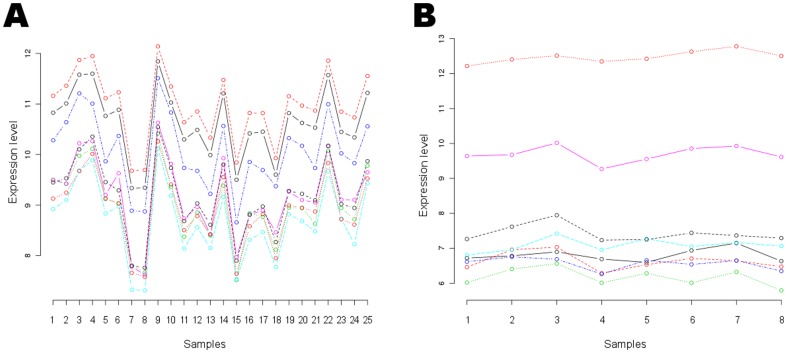
Expression patterns of genes across samples in two types of biclusters. (A) Bicluster containing genes having expression values correlated across the samples. (B) Bicluster containing genes exhibiting a limited variance in the expression values across the considered samples. The X-axis represents the samples included in the bicluster, the Y-axis represents the expression level, and each line shows the expression values of a gene (included in the bicluster) along the various samples of the bicluster.

*Correlation maximization biclustering methods* (CMB) seek for subsets of genes and samples where the expression values of the genes (or respectively samples) correlate highly among the samples (or respectively genes). Figure 1 A illustrates an example of such a bicluster with high correlation between the genes. The algorithm proposed by Cheng and Church [7] searches for this type of biclusters by imposing the condition that the mean square residue is below some parameter 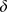. The FLexible Overlapped biClustering (FLOC) technique, proposed by Yang *et al.* [14], is another example of an algorithm belonging to this class.

*Variance minimization biclustering methods* (VMB) search for biclusters in which the expression values have low variance throughout the selected genes, conditions or the whole submatrix. For instance, XMOTIF [15] searches for biclusters with constant gene expressions by imposing the condition that the expression values of each gene are within a very small interval, i.e., each gene exhibits an almost constant expression level for a subset of samples. Another example is the method developed by Hartigan [16], and implemented in several algorithms later on [17], [18]. These methods seek for constant expression values across the selected genes and samples. Figure 1 B illustrates a variance minimized bicluster.

*Two-way clustering methods* (TWC) discover the homogeneous subsets of genes and samples, i.e. biclusters, by iteratively performing one-way clustering on the genes and samples. For instance, the algorithm proposed by Getz *et al.* [19] repeatedly performs one-way clustering on the genes and samples whilst the stable clusters of genes (i.e. clusters of genes that remain constant through the iterations of the algorithm) are used as the attributes for the clustering of the samples, and *vice versa*. Another example is an algorithm proposed by Chun Tang *et al.* [20], which initiates the analysis by clustering the genes to a predefined number of groups (usually 2), and then clusters the samples by featuring each group of genes. Next, the algorithm selects the heterogeneous groups of genes and samples which best represent the distribution of the data, and the whole process is repeated on the selected genes and samples, until the predefined termination condition is satisfied. An example of a termination condition which can be defined by the user is the bicluster size; the algorithm finalizes the analysis once the bicluster size (i.e., number of genes and samples) reaches the threshold.

*Probabilistic and generative methods* (PGM) employ probabilistic techniques to discover genes (or respectively samples) that are similarly expressed across a subset of samples (or respectively genes) in the data-matrix [9], [12], [13], [21]. For instance, the method proposed by Reiss *et al.*, called cMonkey [9], employs Markov chains to model the biclusters. Another example of this method is the probabilistic relational model ProBic [22], which combines probabilistic modelling with relational logic in order to identify the biclusters.

Detailed information regarding the biclustering methods used in our study, including their class, parameters and characteristics, are listed in Tables 1 and 2. Note that when assigning each method to a specific class we prioritized the algorithm over characteristics of the generated biclusters. For instance, FABIA and FABIAS methods [21] are assigned to probabilistic and generative methods (PGM), although they also generate biclusters with low variance (VMB). For each of these methods we also report a list of specifications which are explained in Table 3. In general, there are nine types of parameters that are used by these biclustering methods as detailed in Table 4.

**Table 1:** The class and availability of biclustering methods.

Bicluster Method	Class	Since	Availability	Parameters
ACV [50]	CMB	2007	-	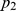 , 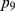
Bayesian Plaid [51], [52]	PGM	2008	C [52]	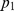
**Bimax ** [**11**]	VMB	2006	Java [53]	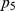
**BiMine ** [**46**]	CMB	2009	Java	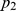 , 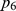
**CC ** [**7**]	CMB	2000	R [54], Java [53]	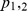
CMonkey [9]	PGM	2006	R	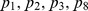
**CTWC ** [**19**]	TWC	2000	MATLAB	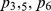
DCC [55], [56]	TWC	2002	-	-
**FABIA and FABIAS ** [**21**]	PGM	2010	R	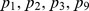
**FLOC ** [**14**]	CMB	2005	R	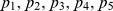
GEMS [57], [58]	CGS	2004	Web, C	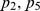
Gibbs biclustering [12]	PGM	2003	-	-
**ISA ** [**43**], [**44**]	TWC	2002	Java [53]	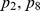
ITWC [20]	TWC	2001	-	-
OP-Clustering [59], [60]	CMB	2003	-	-
**OPSM ** [**40**]	CMB	2003	Java [53], C#	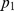
**Plaid ** [**41**], [**42**]	PGM	2002	R [54], web	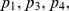
ProBic [22]	PGM	2009	-	-
**QUBIC ** [**45**]	VMB	2009	C	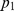 , 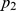 , 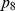
**R/MSBE ** [**30**]	VMB	2006	Java	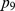
**SAMBA ** [**13**]	PGM	2002	Java [61]	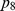
Spectral [62]	VMB	2003	R [54]	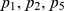
TreeBic [49]	PGM	2010	C	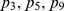
UBCLUST [63]	CMB	2006	Java	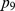
XMOTIF [15]	VMB	2003	R [54], C, Java [53]	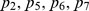
ZBDD [18]	VMB	2005	-	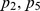
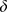 -clustering [16]	VMB	1972	-	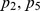
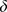 -Pclustering [17]	VMB	2002	-	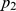
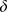 -jk [64]	VMB	2000	-	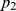 , 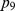

The notations used for the methods classes are stated in the text. The parameters used by the biclustering methods are described in Table 4. The methods that are shown in bold texts were evaluated in our study.

**Table 2:** The biclustering methods specifications and testing data types.

Bicluster Method	Method specifications	Tested data
ACV	GSOVL	Synthetic, yeast
Bayesian Plaid	GSOVL, MCMC, BAYES	Synthetic, yeast
**Bimax**	GSOVL, DISC	Synthetic, yeast
**BiMine**	GSOVL, TREE	Synthetic, yeast
**CC**	GSOVL	Synthetic, Human, yeast
CMonkey	GSOVL, MCMC, MOTIF, TMV	Synthetic, yeast
**CTWC**	GSOVL,SIMA	Human
DCC	NOVL, VECOS	Human
**FABIA and FABIAS**	GSOVL, EM, BAYES, SVD	Synthetic, Human
**FLOC**	GSOVL, TMV	Synthetic, Human
GEMS	GSOVL, MCMC	Synthetic, Human
Gibbs biclustering	GSOVL, DISC, MCMC, BAYES	Synthetic, Human
**ISA**	GSOVL	Synthetic, yeast
ITWC	SOVL, VECOS	Human
OP-Clustering	GSOVL, TREE	Yeast, Human
**OPSM**	GSOVL, DISC	Synthetic, yeast [43], Human
**Plaid**	GSOVL, FUZZY [31]	Synthetic, Human, yeast
ProBic	GSOVL, EM, BAYES, TMV	Synthetic, yeast
**QUBIC**	GSOVL	Synthetic, yeast, e. coli, Human
**R/MSBE**	GSOVL	Synthetic, yeast
**SAMBA**	GSOVL, DISC	Yeast, Human
Spectral	NOVL,SVD	Human
TreeBic	GSOVL, MCMC, BAYES, TREE	Human
UBCLUST	GSOVL, DISC, MCMC, SIMA	Synthetic, yeast
XMOTIF	GSOVL	Synthetic [43], Human, yeast [43]
ZBDD	GSOVL	Synthetic, yeast
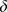 -clustering	NOVL	Synthetic
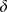 -Pclustering	GSOVL	Synthetic, yeast
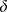 -jk	GSOVL	Synthetics, Human

The methods specifications are described in Table 3. Although the original FLOC algorithm is tolerant to missing values (TMV), the R implementation available in BicARE (V 1.2.0) of the Bioconductor package does not accept missing values in input data. Note that all the tested data with missing citations were studied by the developers of the algorithms to which they have been assigned. For the citation of the algorithms see Table 1.

**Table 3:** Various specifications considered for the biclustering methods.

Specifications	Description
GOVL	The obtained biclusters are allowed to have overlaps over only the gene-sets.
SOVL	The obtained biclusters are allowed to have overlaps over only the sample-sets.
GSOVL	The obtained biclusters are allowed to have overlaps over both gene and sample-sets.
NOVL	No overlaps at all are allowed for the obtained biclusters.
DISC	Discretization is mandatory for running the algorithm
TMV	The method is tolerant to missing values.
SIMA	Simulated annealing is applied to avoid convergence to local optima.
VECOS	Vector Cosine Scores is applied to measure the similarities of the samples (or genes).
SVD	The method applies a form of Singular Value Decomposition.
MCMC	The method employs a Markovian Chain Monte Carlo approach.
BAYES	The method employs a fully Bayesian approach.
EM	The method uses the Expectation-Maximization method.
MOTIF	The MOTIF sequence co-occurrence is considered in the biclustering approach.
TREE	The method applies a tree structure for discovering suitable sets of genes and samples.

**Table 4:** Different types of parameters used by the biclustering methods.

Parameter	Parameter specification
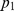	the number of generated biclusters either per iteration or globally
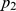	the threshold for biclustering optimization criteria
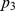	the threshold for the number of iterations
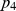	the probability of including/excluding a gene or a sample during the clustering process
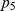	the threshold for the size of the biclusters
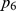	the threshold for the number of gene (or respectively sample) operations in one iteration
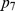	the number of genes and/or samples in the initial bicluster seeds)
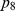	the overlap threshold for the obtained biclusters
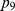	model-based parameters, e.g., parameters for prior distributions, or tree depth

The operations allowed when defining parameter 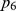 are comparisons, additions, removals, and splits for genes (or respectively samples).

### Experiment setup

The multi-tissue data we use within our study consists of 228 samples from 5 distinct healthy human tissues from the GeneSapiens database [23]: 59 blood t-cell, 95 cerebral cortex, 13 liver, 41 striated muscle, and 20 testis samples. The selected tissues are transcriptionally distinct and clearly defined, hence featuring a minimal risk of annotation errors. GeneSapiens contains Affymetrix based human gene expression data collected from publicly accessible biological sources, namely Gene Expression Omnibus and ArrayExpress. It includes 175 different cancer and tissue types with altogether over 130 million data-points. To construct GeneSapiens, data from CEL files of different types of Affymetrix microarray generations were normalized together in a specifically developed three-step process (Kilpinen et al [23], Autio et al [24]) to create a large integrated data collection across different studies and array generations. Using the selected data we constructed a gene expression matrix with 11834 rows and 228 columns corresponding to the considered genes and samples, respectively. In the end all genes with missing expression values were excluded from the gene-expression matrix.

To create the breast tumor data gene expression microarrays were downloaded from The Cancer Genome Atlas for primary breast carcinoma tumors and controls. First, probes matching either multiple or no genes were removed. Then, data were normalized to a mean of 0. The original data can be obtained from TCGA web site http://cancergenome.nih.gov/. The TSP study accession number of the raw data in the database of Genotype and Phenotype (dbGaP) is phs000569.v1.p7. Two clinically well-defined subgroups of breast tumor (ER+/PR+/HER2+ and ER−/PR−/HER2−) and healthy breast samples were chosen for our analysis. All genes with a variance less than one across the samples were also discarded.

### Quality Evaluation Benchmarks

Recently, K. Eren and colleagues studied a collection of biclustering methods on several synthetic data matrices that housed various types of bicluster structures and estimated how well each method discovers them [25].

In addition to running time analysis, they reported results of Gene Ontology based enrichment analysis in order to evaluate the gene-sets of biclusters discovered in a gene-expression data of Rat peripheral and brain regions. Here we, however, focus on the biological relevance of the biclusters discovered by the 13 biclustering and 2 one-way clustering methods. In particular, we focused on the ability of these methods to distinguish various sample types rather than their performance in discovering various bicluster patterns in the data. In this regard, we consider four kinds of benchmarks: one sample-based, two gene-based and the running time. All the applied benchmarks measure how much the generated clusters succeed in incorporating *a priori* knowledge. These benchmarks can be classified as external benchmarks as described by Santamaria *et al.* [26].

#### Sample-based benchmarks

Sample-based benchmarks evaluate the (bi)clusters generated by a given method by assessing the set of samples included in them. These benchmarks answer the question of how well a method can distinguish different types of samples. If we denote by 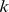 the number of different types of samples (e.g. blood T cell or liver samples in the multi-tissue data that we use), then let 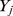, with 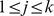, denote the sub-matrix which contains all the rows from the original data matrix but only those columns which are associated to the samples of type 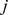. We also denote by 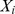 the 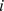-th bicluster generated by a given biclustering method, by 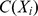 and 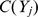 the set of columns included in the two sub-matrices 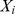 and 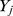, respectively and by 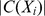 and 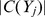 the number of elements in these two sets. Then, the formula
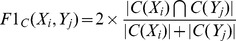
(1)characterizes the level of overlap between the sets of columns of the two submatrices 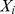 and 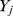. In particular, Equation (1), which is based on Sørensen similarity [27] and Dice's coefficient indices [28], returns a value in the range [0,1], with 1 indicating that the set of columns of the bicluster 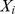 includes the whole set of samples of type 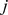, and 0 meaning that 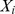 does not contain any of the samples of type 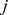.

Equation (1) allows to define, for each biclustering method, a matrix 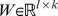, where 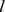 and 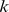 are the number of generated biclusters and the number of distinct sample types considered, respectively. Each entry 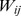 is the value 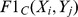 representing the coverage of the samples of type 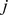 by the columns of the 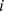
th generated bicluster. Then, we construct a vector 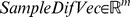, where 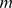 is the minimum of the indices 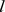 and 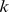, that describes how well the biclustering method has distinguished different sample types. This vector is actually obtained through an iterative greedy approach where the maximum value of the matrix 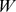 is first extracted and then its corresponding row and column are deleted. The procedure continues to extract the maximum value of the remaining data and then to remove the related rows and columns until no row or column remains. At the end of this process, we collect all the extracted maximum values within the vector 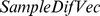. The mean of the values in 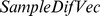 is considered as the quality measurement for the biclustering method, i.e., the *sample differentiation benchmark SampleDif*. Note that contamination of the samples with other tissue types or miss-annotation of the samples can affect the sample differentiation.

#### Gene-based benchmarks

This category refers to those benchmarks that estimate the quality of the (bi)clusters by assessing the genes included in them. Here we consider two such benchmarks.

**Figure 2:**
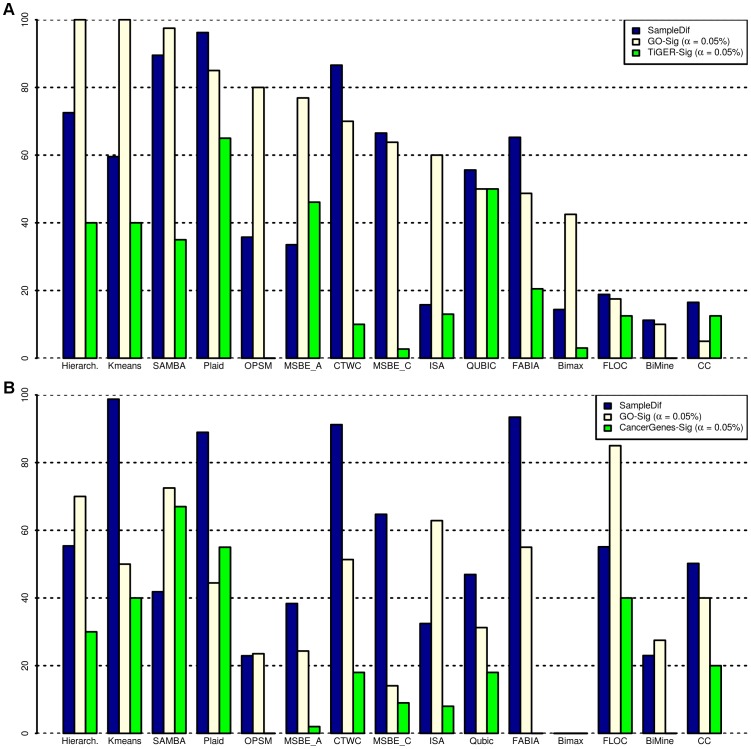
Sample-based (i.e. sample differentiation) and gene-based benchmarks (i.e. GO-Sig and TiGER-Sig) for thirteen biclustering and two clustering methods for the Multi-tissue type (A) and the breast tumour (B) data.

*Gene Ontology-based significance* (denoted by *GO-Sig*) is one of the widest used gene-based benchmarks for biclustering methods [11], [25], [29]–[33]. It indicates how significantly the sets of genes discovered by a biclustering method are enriched with a similar GO category provided by the Gene Ontology Consortium [34]. To estimate this, we used the FuncAssociate 2.0 webtool provided by Berriz *et al.* [35]. Initially, Fisher's exact test [36], was used to estimate a p-value which could be described as the probability of a GO category being equally or more frequently observed if we randomly pick the same number of genes as those included in a given bicluster. Next, an adjusted p-value is estimated by using the Westfall and Young procedure [37] with 1,000 re-samplings. Finally, for each biclustering method, we set its GO-based significance to be the percentage of the generated biclusters featuring adjusted p-values less than parameter 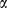. For our analysis we chose the threshold 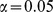, see Figures 2A and 2B.

*TiGER-based significance* (denoted by *TiGER-Sig*) indicates the percentage of the biclusters generated by each method that include genes specific to the studied sample-types. For the multi-tissue type gene expression data we employ the Tissue-specific Gene Expression and Regulation (TiGER) database [38], which is constructed based on the known tissue-specific genes, TFs and cis-regulatory modules. The database includes 7,261 tissue-specific genes, which were discovered after analyzing the expression patterns of approximately 54,000 genes among 30 various human sample-types. In particular we were interested in those tissue-specific genes that are associated with our selected sample types: blood t-cell, cerebral cortex, liver, striated muscle, and testis. That is, we analyzed how well the studied biclustering and clustering methods can identify these genes. To do this, we apply a symmetric version of the formula 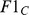 in which we look for the overlap of the gene-sets instead of the sample-sets, see equation (2).
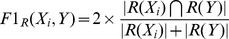
(2)The submatrix Y now contains all the columns of the initial matrix and only those rows which correspond to the genes that are specific to the tissue types considered in the multi-tissue type gene expression data. Then, we denote by 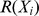 and 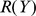 the sets of rows included in the two sub-matrices 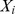 and 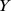, respectively and by 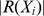 and 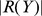 the number of elements in these two sets. Thus, the formula 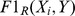 indicates the level of overlap between the sets of rows of the two sub-matrices 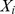 and 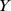, i.e., the coverage of the genes specific to all sample types considered here by the 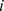
th generated bicluster. Then, for each biclustering method, if we denote by 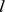 the number of generated biclusters, we compute an 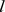-dimensional vector with all its entries in the range [0, 1]. The values in this vector are obtained by using formula (2) and the mean of these values indicates how well the biclusters extracted by the algorithm cover the genes specific to our samples. We also investigated whether similar or higher overlap values could be obtained by randomly selecting genes from the gene-expression data. To do this, we computed a p-value for each of the generated biclusters with 1,000 re-samplings (similarly to the second phase of the GO-based significance). The p-value is the proportion of the 1,000 randomly picked genes that have higher overlaps with the genes specific to the selected sample types, compared with the genes discovered by the biclustering methods. Finally, for each biclustering method, we set its TiGER-based significance to be the percentage of the generated biclusters that feature a p-value less than parameter 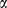. In our analysis we chose 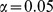, see Figures 2A and 2B. For the Breast cancer gene-expression data we applied the exact same method that was described for the multi-tissue type data except that instead of the Tissue-specific Gene Expression and Regulation (TiGER) database we used CancerGenes [39]. CancerGenes provides cancer related genes that have been retrieved from several gene-based resources, e.g. NCBI Entrez Gene, Ensembl BioMart, and Sanger COSMIC, and their relevant annotations such as functional description of the genes, the gene locations, Entrez Gene ID, GO terms, InterPro descriptions, gene structure, and experimentally determined transcript control regions. Note that as mentioned previously, the gene-based evaluation methods are external benchmarks hence their results are dependent on the quality and the completeness of the database that they use. As for instance, since the GO-sig is dependent on the GO categories provided by the Gene Ontology Consortium [34] changes in the data-base can affect the GO-Sig results. Moreover, large overlaps on the genes of the biclusters extracted by an algorithm can bias the GO-Sig results in favoring these algorithms.

#### Running time

In addition to the quality of the extracted (bi)clusters, it is also important that the analysis is done in a reasonable amount of time. Thus, we compared the running time of the studied algorithms.

Using these four benchmark measures we evaluated 13 biclustering methods: SAMBA [13], OPSM [40], Plaid [41], [42], Additive and Constant MSBE [30], ISA [43], [44], CTWC [19], BiMax [11], FABIA [21], QUBIC [45], FLOC [14], CC [7], BiMine [46], as well as the two most popular one-way clustering methods, *k*-means [47] and hierarchical [48]. All these methods were able to extract at least one (bi)cluster from our data. Note that in addition to the mentioned methods, we also executed Treebic [49] on our data but after running of the algorithm no biclusters were discovered from any of our data. In this respect, we will only report the running time of the algorithm in the results section.

### Parameter settings

The Euclidean distance metric was used for the *k*-means method and the Pearson distance for the hierarchical method. The cluster number threshold for both was also set to 10 when clustering the genes. For clustering of the samples the threshold was set to 5 for the multi-tissue type gene-expression data and 3 for the breast tumor. Moreover, within the hierarchical clustering we used the complete linkage method. In SAMBA the overlap prior factor was set to 0.1. The responding probes to hash was set to 100 and the hash kernel size (minimal and maximal) was set to 4. The hash-tables are the data structures used by the algorithm to store the converging biclusters (i.e. weighs of the edges of a bipartite graph in which the nodes represent a selection of genes and samples)[13]. The number of the accepted biclusters in each iteration for the OPSM method was set to 10. For the Plaid model the row and column release probabilities were set to 0.7 and the maximum number of layers to 40.

In the additive and constant MSBE biclustering methods the 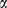 parameter (the threshold for the applied similarity score) was set to 0.4, 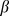 (the bonus for the similarity score) to 0.5, and 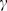 (the quality and size threshold of the biclusters) to 1.2. The ISA method was run on 100 initial points, with gene and sample score thresholds set to 2. The parameters for the CTWC method were set as follows: the minimum gene size was set to 15 while the sample size was set to 5. The minimum size of the genes and the samples of the Bimax biclusters were set to 2. We ran the FABIA method to achieve 40 biclusters while the other parameters were set to their default values. We ran BiMine with minimum sample size of 13 and the threshold for the Average Spearman's 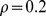. The residue threshold for the FLOC method was set to 0.01, the sample and gene initial probabilities were set to 0.4, the minimum sample size of a bicluster was set to 13 and the minimum gene size was set to 15. The 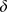 parameter was set to 50.0 and 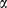 to 1.5 for the CC algorithm. Moreover, the CC algorithm was set to extract 40 biclusters. The parameters for the QUBIC method were set to their default values i.e., the quantile discretization was set to 0.06, the number of ranks and filtering overlapping blocks were set to 1, minimum sample size was set to 2, the conservation parameter of the blocks was set to 0.95 and the number of the reporting blocks was set to 100. Bicluster results with sample or gene sizes smaller than 10 were ignored in our analysis. The number of bicluster results for methods that extracted large number of biclusters, e.g. BiMine (4301 biclusters) and SAMBA (102 biclusters), was limited to 40. After ignoring bicluster results with gene and sample sizes less than 10, the biclusters for each of these methods were sorted in decreasing order based on their column size (number of discovered samples) and the top 40 were chosen for further study. This filtering simplified the analysis by limiting the number of the results and also improved the results by excluding the smaller size biclusters which either highly overlap larger biclusters or their size of samples or genes are too small to detect any reliable gene expression patterns.

## Results

The results for the sample-based and gene-based evaluations of the 13 biclustering and 2 clustering methods on the multi-tissue type data and the breast tumor data are illustrated in Figures 2A and 2B, respectively. The biclustering and clustering methods were chosen based on their availability, ease of installing and execution, and also based on the fact that they were able to find at least one (bi)cluster in our datasets. All values were converted to percentage scale. Given that the most common evaluation method for the biclustering algorithm is the GO-Sig and the main goal of most biclustering algorithms is to identify gene-sets that are co-expressed across a subset of samples rather than differentiating the sample-types, in Figure 2A we have ordered the bicluster algorithms based on their GO-Sig values. Moreover, to simplify the comparison, we used the same order of biclusters in Figures 2A and 2B. Here we first describe how the biclustering methods performed in the multi-tissue type data and then describe how the performance values changed in the breast tumor data.

### Multi-tissue type data

For the heterogeneous data, three biclustering methods feature sample differentiation values larger than 80%: Plaid (96.2%), SAMBA (89.5%), and CTWC (86.5%), as shown in Figure 2A. This indicates that these methods are able to distinguish the particular sample types in the multi-tissue type data. The sample differentiation values given by the hierarchical clustering (72.5%) and *k*-means (59.5%), as well as those given by constant MSBE and FABIA biclustering methods, were also relatively high (∼60%).

The GO enrichment analysis indicated that *k*-means (100%), hierarchical (100%), and SAMBA (97.5%) generated a high percentage of gene sets that were significantly annotated. Additionally, a relatively high proportion of the OPSM (80%) and Plaid (85%) biclustering results were also enriched. The TiGER-Sig analysis also showed that Plaid (65%), QUBIC (50%), additive MSBE (46.1%), hierarchical clustering (40%), and *k*-means (40%) algorithms discover gene-sets significantly enriched with genes specific to the studied samples.

### Breast tumor data

The sample differentiation and the GO-Sig measurements of the biclustering methods ISA, FABIA, FLOC, CC, and BiMine were clearly improved for the more homogenous Breast tumor data comparing to their performance for the heterogeneous multi-tissue type data, see Figures 2A and 2B. In contrast, the GO-Sig of the two conventional clustering methods (*k*-means and heirarchical) were decreased. However, as opposed to the Hierarchical clustering the *k*-means differentiated the two breast tumor subtypes (ER+/PR+/HER2+ and ER−/PR−/HER2−) and the healthy breast samples accurately (99%). FABIA (93%), CTWC (91%), and Plaid (89%) biclustering methods also differentiated the various cancer sub-types very well. The 5 methods that discovered gene-lists in which the highest percentage feature significant common GO annotations are FLOC (85%), SAMBA (73%), Hierarchical (70%), ISA (63%), and FABIA (55%). Moreover, more than half of biclusters discovered by SAMBA (67%) and Plaid (55%) significantly overlapped genes that were reported by the CancerGenes to be related to cancer. When we executed Bimax on the breast tumor data the algorithm did not converge in a reasonable time (720 hours) hence we could not extract any biclusters from the data. In addition to estimating the fraction of the (bi)clusters that featured similar GO annotations, we also studied the significantly common GO categories that were extracted by GO analysis of the bicluster results. We found that gene-lists discovered by the CTWC, FABIA, ISA, Plaid, SAMBA, and hierarchical clustering were significantly enriched with GO terms: cell cycle, M phase of the cell cycle, mitosis, cell division, proliferation, and response to stress. Moreover, gene-lists discovered by CTWC, FABIA, ISA, and SAMBA were annotated with immune response. A subset of these GO categories (i.e. cell cycle, M phase and immune response) were also reported by Wang *et al.* [8] as the results of GO analysis on biclusters extracted from breast tumor data. The gene-lists discovered by other (bi)clustering methods that we studied were annotated to a smaller subset of the mentioned GO terms.

### Running time

**Figure 3:**
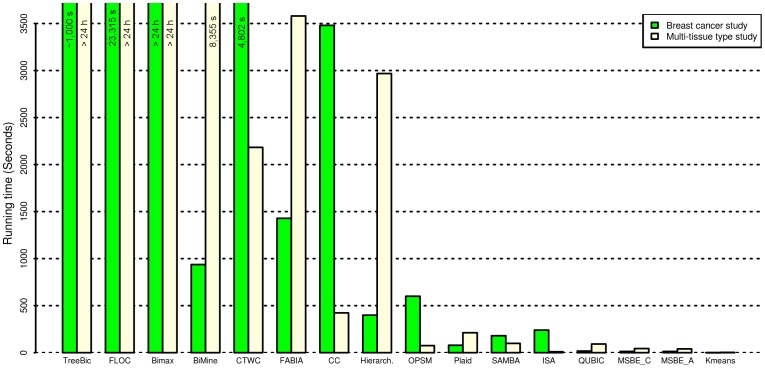
Running time of the thirteen biclustering and two clustering methods on the Breast cancer microarray and multi-tissue type microarray.

The running time of the 13 biclustering and 2 clustering algorithms that worked successfully on our two micro-array data are illustrated in Figure 3. The algorithms were executed on a computer with Intel Quad CPU (Q9650), 15.6 GB memory and operating system Ubuntu 10.04 LTS (the Lucid Lynx). However, since we could not run the software package for QUBIC locally, we used the online application on their servers. In ascending order and based on the mean of the amount of time that took each algorithm to extract (bi)clusters from the 2 data, the 8 methods that ran in less than 10 minutes (600 secs) are: *k*-means clustering method (3 secs for multi-tissue type data, and 0.8 secs for breast tumour subtype data), MSBE-Additive (40 secs, 13 secs), MSBE-Constant (44 secs, 13 secs), QUBIC (92 secs, 17 secs), ISA (10 secs, 240 secs), SAMBA (99 secs, 180 secs), Plaid (212 secs, 78.8 secs).

## Discussion

Clustering is a powerful approach to extract biologically relevant information from the high-throughput data. While clustering techniques, such as k-means or hierarchical clustering, are able to find similarities of genes over all conditions (or conditions over all genes), biclustering methods search for local patterns that may feature important biological or medical implications. Here we have compared 15 (bi)clustering methods by analyzing different aspects, such as their approach and parameter settings. Moreover, we have introduced several evaluation measures for comparing the performance and application of these biclustering methods.

Our results show that Plaid, SAMBA, CTWC, hierarchical clustering, constant MSBE, and FABIA methods best distinguished the various sample-types in the multi-tissue type gene expression matrix. Moreover, the GO enrichment analysis indicated that the gene-sets generated by the *k*-means, SAMBA, hierarchical clustering, OPSM, and Plaid methods were significantly annotated with similar Gene Ontology categories when they were applied on the multi-tissue type data. However, OPSM discovered biclusters with relatively high mean overlap on their genes (55%). This can bias the GO-Sig results in favoring OPSM algorithm. The TiGER-Sig analysis on the multi-tissue data also confirmed that the Plaid, QUBIC, additive MSBE, hierarchical clustering, and *k*-means discovered gene-sets significantly enriched with genes that are specific to our studied samples. The high performance of the one-way clustering methods on the multi-tissue data was expected since the heterogeneity of the samples can favor methods that extract non-overlapping sets of genes or samples from the data (e.g. *k*-means and hierarchical clustering). On breast cancer data, *k*-means best differentiated the two breast tumor subtypes (ER+/PR+/HER2+ and ER−/PR−/HER2−) and the healthy breast samples. FABIA, CTWC, and Plaid differentiated the samples almost as good as the *k*-means. The gene-sets generated by FLOC method were also most frequently enriched with similar GO categories in the breast tumor data analysis. However, similar to OPSM in multi-tissue type data analysis, we believe that the high GO-Sig of this method is biased by the high mean overlap (55%) of the genes discovered by FLOC. A considerable fraction of the results generated by SAMBA, hierarchical and ISA were also significantly annotated with similar Gene Ontology categories. Taken together, we found that no single method performs the best in all measurements and on both data.

When comparing the performance of the (bi)clustering methods on the two data sets of our study we realized that in the more homogeneous breast tumor data the GO-sig of the two conventional clustering methods (and the sample differentiation of the hierarchical clustering) have decreased. Also, when applied on the breast tumor data set (with more homogeneous samples comparing to the multi-tissue type data) all benchmarks for CC, FLOC, and BiMine (and sample-differentiation and GO-Sig of FABIA) increased while all benchmarks for the Qubic, MSBE-A, MSBE-C and Plaid decreased. It is worth mentioning that, except FABIA, all the biclusters with improved performance (i.e., CC, FLOC, BiMine) were members of the CMB (Correlation Maximization Biclusters) class. FABIA seeks for Variance Minimized Biclusters although classified as PGM because of its use of probabilistic and generative models. The methods with declined performances were of different classes: Qubic, MSBE-A and MSBE-C methods are VMB (Variance Minimization biclusters); The Plaid model is PGM (Probabilistic and Generative Methods); and OPSM is CMB.

Our results are in line with other biclustering comparison studies. For instance, Hochreiter *et al.* [21] developed the method FABIA and used the Jaccard index as the similarity measurement in combination with the Munkres algorithm to estimate the sample differentiation. They used three data sets for testing and their results are similar to ours: in multi-tissue type data set, Plaid not only distinguishes sample types better than FABIA, but differentiates the samples better than all their studied biclustering methods. Moreover, when they run the algorithms on breast tumor data the situation reverses and FABIA performs better than Plaid. All these were also observed in our results. In another study, K. Eren *et al.* [25] reported that when running a collection of biclustering methods on a data set constructed of rat peripheral and brain regions samples, a high fraction of the biclusters generated by the Plaid method and a low percentage of those generated by the Bimax seem to feature similar GO annotations compared to other available methods.This result is in line with our multi-tissue type data analysis. Overall, Plaid performed robustly when tested on Breast tumour (GDS3716), Human skeletal muscles GDS3715, C blastomere mutant embryos (GDS1319), Rat lung SM exposure model GDS1027, Rat peripheral and brain regions GDS589 studied by K. Eren et al.; and performed equally good when executed on the multi-tissue data and breast tumour samples studied by S. Hochreiter et al. As mentioned previously, Plaid together with SAMBA also performed acceptable in the multi-tissue and breast tumour samples that we studied.

To conclude, taking into consideration our analysis and the results reported by K. Eren et al. and S. Hochreiter et al. as well as the limitations and pitfalls of the evaluation methods, biclustering methods such as Plaid and SAMBA are useful for extracting relevant subsets of genes and samples from microarray experiments as long as the samples are well defined and annotated, the contamination of the samples is limited, and the samples are well replicated. Moreover, our results indicate that biclustering algorithms such as Plaid and SAMBA find more relevant gene-sets comparing to the clustering algorithms when the samples are not highly heterogeneous. This suggests that in studies where different samples feature common active biological processes and genes are also active in several biological processes (e.g. cancer studies), biclustering algorithms could discover more relevant genes comparing to one-way clustering methods.
